# Cracks Reinforce the Interactions among Soil Bacterial Communities in the Coal Mining Area of Loess Plateau, China

**DOI:** 10.3390/ijerph16244892

**Published:** 2019-12-04

**Authors:** Zhanbin Luo, Jing Ma, Fu Chen, Xiaoxiao Li, Huping Hou, Shaoliang Zhang

**Affiliations:** 1 School of Environment Science and Spatial Informatics, China University of Mining and Technology, Xuzhou 221008, China; lzbin1991@cumt.edu.cn (Z.L.); lixiaoxiao@cumt.edu.cn (X.L.); houhuping@cumt.edu.cn (H.H.); slzhang@cumt.edu.cn (S.Z.); 2 College of Resources and Environment, Northwest A&F University, Yangling 712100, China; 3 Low Carbon Energy Institute, China University of Mining and Technology, Xuzhou 221008, China; jingma2013@cumt.edu.cn

**Keywords:** soil bacterial community, molecular ecological network, coal mining, Loess Plateau

## Abstract

Soil microorganisms play a key role in global biogeochemical changes. To understand the interactions among soil bacterial communities and their responses to extreme environments, the soil properties and bacterial community diversity were determined in the post-mining ecosystem of the Loess Plateau, China. The results showed that the soil temperature, pH, organic matter, available phosphorus, and available potassium values were significantly reduced in the post-mining cracks area. However, the richness and uniformity of soil bacterial communities increased by about 50% in the post-mining cracks area. Soil microbial community structure and the network interactions tended to be complex and strengthened in the post-mining cracks area. Moreover, soil nutrient loss caused the differences in soil bacterial community structure compositions in the post-mining cracks area. Furthermore, the relationships between soil physicochemical properties and different modules of the soil bacterial molecular ecological network were changed in a complex manner in the post-mining cracks area. This study provides a theoretical basis for adaptive management and response to cracks in post-mining areas and under other extreme conditions.

## 1. Introduction

The mining industry is an important anthropogenic activity worldwide [1,2]. The rapid development of the mining industry has promoted the progression of human society and civilization [3,4]. Approximately 3.6 billion tons of coal are produced each year in China, which accounts for 55% of the global yield [1]. Due to the high-intensity coal mining disturbances, a series of ecological environment problems have been introduced [5,6,7]. The quantity of damaged land is more than 48,000 hectares every year in China [8]. Therefore, more attention should be paid to environmental issues and sustainable socio-economic development in post-mining areas [5,6,9,10].

Mining activities have damaged the surface ecosystem [6]. Coal mining cracks (or fissures) represent an important type of coal mining disturbance that occurs during the mining process, especially in the fragile ecosystem of the Loess Plateau [11,12,13,14,15]. Under the triple pressure of harder soils, water deficiency, and high-intensity underground coal mining, cracks are more easily formed with different depths and widths [16,17]. In previous decades, numerous studies have explored the observational approaches and developmental features of mining-induced ground fissures [18,19,20,21]. Moreover, some studies have focused on the effects of coal mining cracks on soil physicochemical properties [22,23,24,25,26,27]. They have found that soil water, temperature, and bulk densities decreased [22,23,24]. Additionally, they have reported that the elements nitrogen, phosphorus, and potassium were lost [25,26,27]. Furthermore, some studies have reported that the environment for the vegetation root system was destroyed, causing the vegetation to wither and die on the ground [28,29,30]. However, there is little understanding on how coal mining cracks have changed the soil bacterial community structure and its interactions. Fortunately, a number of studies have evaluated the effect of land reclamation on soil bacterial communities after coal mining disturbance in recent years [31,32,33,34,35,36,37]. Most studies have observed that the soil bacterial community diversity and activity of reclaimed lands were higher than that of non-reclaimed soil in mining area [32,33,34,35,36,37]. Moreover, some studies have focused on the relationship between the changes in soil bacterial communities and surrounding environmental factors [31,32,36,37]. They have found that the changes in soil bacterial communities are closely related to soil physicochemical properties [37,38], enzyme activities [32], and types of vegetation cover [31,34].

Based on the high-throughput sequencing datasets, as a new method, molecular ecological network analysis could provide new insights for revealing microbial interactions in the natural ecological environment [39,40,41,42,43]. A growing number of studies have succeeded in using this tool for analyzing the microbial interactions in different ecological systems [39,44,45,46,47,48,49,50,51]. For example, some studies have observed that soil microbial molecular ecological networks change at different levels of elevated CO_2_ [39,49,51]. Some studies have found that temperature is the best predictor for microbial community variations in the timberline [52,53,54]. Moreover, another study showed that higher precipitation strengthened the microbial interactions in semi-arid grassland soils [55].

To determine the effects of coal cracks on the changes in soil bacterial interactions, molecular ecological network analysis was used to construct networks for two groups, namely the coal mining crack zone and the non-crack control area in the Loess Plateau. Furthermore, the relationships among the key soil physicochemical properties and soil bacterial communities were also explored. This study might enhance our understanding of the network interaction changes and the emergency responses of soil bacterial communities and provide a basis for further research into coal mining disturbance areas globally.

## 2. Materials and Methods

### 2.1. Study Area and Soil Sampling

**Figure 1:**
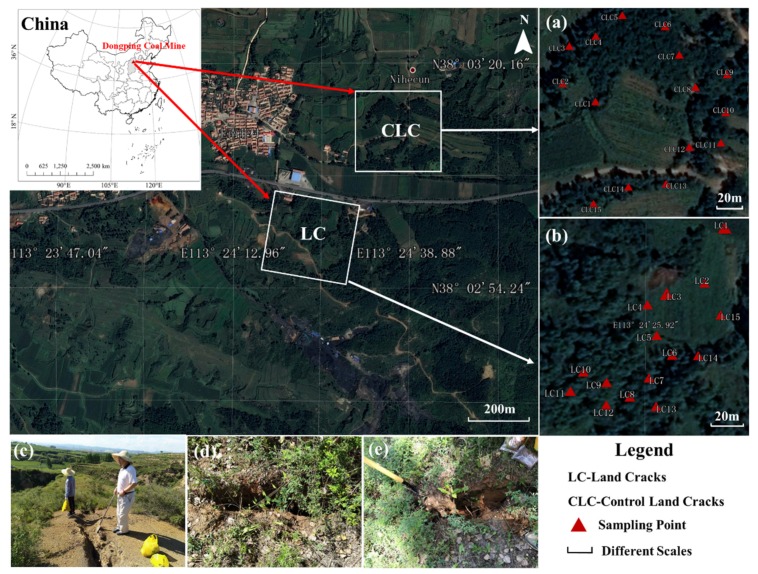
Location of the study area, soil sampling sites, and ecological investigation. (**a**) Soil sampling of the control area, Control Land Cracks (CLC); (**b**) soil sampling of land cracks area, land cracks (LC); (**c**) field survey of land cracks; (**d**) land cracks caused by coal mining; (**e**) collection of soil samples from land cracks.

The Dongping Coal Mine (113°15′ E–113°18′ E, 38°01′ N–38°03′ N) is located in Yangquan City, Shanxi Province, China. It has a continental climate with an annual average temperature of 8.7 °C and an annual rainfall of between 450 and 550 mm. The region is at the south of the Loess Plateau, and the main soil type is calcareous cinnamon soil. The Dongping Coal Mine area has rich coal resources, with a mineral area of 16,242 km^2^ and a proven geological reserve of 134,310,000 tonnes. The annual approved mining capacity is 1,200,000 tonnes. However, the ecological environment has been seriously damaged with frequent land cracks and exposed vegetation root systems at the surface (Figure 1).

During 17–18 August 2018, a total of 30 soil samples were collected. In the land cracks area, surface soils (0–20 cm) were collected along the investigation direction using the serpentine dot patterns method. Fifteen coal mining cracks, which formed by coal mining face disturbance in 2015–2017 in Dongping Coal Mine, were selected as the sampling locations. All of the sampling points were covered by shrubs and grasses. Five sub-samples were taken from each crack and mixed into one sample. These parts of the soil samples are marked as LC (Land Cracks, LC1–LC15) (Figure 1a). In the control area, surface soils (0–20 cm) were collected along the investigation direction using the same method on the land cracks area. The control sampling position was not affected by coal mining disturbance that was outside the working face. There were no cracks, but there were similar landscapes to the land cracks area that were covered by some shrubs and grasses. Five sub-samples were taken from each point and mixed into one sample. These parts of the soil samples were marked as CLC (Control Land Cracks, CLC1–CLC15) (Figure 1b). To avoid the contamination of soil samples, we wore sterile gloves and collected the surface soil samples of the unstained sampling shovel during the sampling process. These soil samples were packed into the clean and appropriative PVC bags and stored at 4 °C in a car refrigerator. Then, they were quickly returned to the laboratory for soil processing. In the laboratory, some parts of the soil samples were dried indoors and gravel, plant residues, and other impurities were removed. After grinding over a 2 mm sieve, these soil samples were used to test the soil physicochemical properties. Other fresh soil samples were stored at −20 °C and used for soil microecological diversity sequencing [56].

### 2.2. Processing of Soil Physicochemical Properties

Soil water (W) and soil temperature (T) were determined using a soil temperature and humidity meter (TR-6, Guandong Shunkeda, Guangzhou, China) when collecting soil samples by the Mining Area Ecological Survey Group [56]. Soil pH values were measured using the pH meter (PHC-3C, Shanghai Leici, Shanghai, China) with a ratio of soil and water of 1:5 [57]. Soil electrical conductivity (EC) values were determined using the laboratory conductivity meter (DDS-307A, Leici, Shanghai, China) [57]. Soil organic matter (OM) content was measured using the potassium chromate oxidation colorimetric method [57]. The available phosphorus (AP) content was determined by the hydrochloric acid ammonium chloride method [57]. The soil nitrate nitrogen (NN) content was determined by the calcium chloride colorimetry method [51]. The soil ammonium nitrogen (AN) content was measured by the acid-soluble molybdenum antimony colorimetric method [21]. These contents of OM, AP, NN, and AN were measured using an UV-VIS (Ultraviolet–visible) spectrophotometer (Specord 210Plus, Analytik Jena AG, Jena, Germany). The soil available potassium (AK) content was measured using the ammonium acetate extraction colorimetric method with an atomic absorption spectrophotometer (TAS-990, Persee, Beijing, China) [58].

### 2.3. DNA Extraction, PCR Amplification, and Illumina Miseq Sequencing

DNA extraction was done using the FastDNA™ SPIN Kit for Soil (MP 112 Biomedicals, Solon, OH, USA) from 0.5 g fresh soil samples. DNA extraction quality was determined by 0.8% agarose gel electrophoresis, and the concentrations were quantified by an UV-VIS (Ultraviolet–visible) spectrophotometer (Specord 210Plus, Analytik Jena AG, Jena, Germany). PCR (Polymerase Chain Reaction) amplification was carried out at the V4–V5 regions of 16S rRNA genes of the bacteria, where the primer set was 515F (5′-GTGCCAGCMGCCGCGGTAA-3′) and 907R (5′-CCGTCAATTCMTTTRAGTTT-3′). The reaction components of the PCR amplification reaction system (25 µL) were as follows: 5× reaction buffer (5 µL), 5 × GC buffer (5 µL), 2.5 mM of dNTP (0.5 µL), 10 µM of Forward primer (1 µL), 10 µM of Forward primer (1 µL), DNA Template (2 µL), Q5 DNA Polymerase (0.25 µL), and Dd H_2_O to 25 µL. The amplification conditions were as follows: initial denaturation at 98 °C for 2 min, denaturation at 98 °C for 15 s, annealing at 55 °C for 30 s, extension at 72 °C for 30 s, final extension at 72 °C for 5 min, and cooling hold at 10 °C. Then, the triplicate applications were pooled together, electrophoresed on a 2% (*w*/*v*) agarose gel, and recovered by an AxyPrep DNA Gel Extraction Kit (50prepAP-GX-50, AXYGEN, Wuhan, China). Fluorescence quantitative analysis of the above products was done using Quant-iT PicoGreen dsDNA Assay Kit (P7589, Invitrogen, Carlsbad, USA) in the Microplate reader (FLx800, BioTek, Vermont, USA). Then, the composite sequencing library was constructed by combining equimolar ratios of amplicons from all 30 samples. Finally, the 30 sample libraries were analyzed by using the platform of MiSeq PE250 from the Personal Biotechnology Company, Shanghai, China [51].

### 2.4. Bioinformatics and Statistical Analysis

Using FLASH (Fast Length Adjustment of short reads, v1.2.11, http://ccb.jhu.edu/software/FLASH/), QIIME (Quantitative Insights into Microbial ecology, v2018.11, http://qiime2.org/), and USEARCH (Ultra-fast Sequence Analysis, V11.0.667, http://www.drive5.com/usearch/), the sequencing data were preprocessed. Excel and SPSS 22.0 software (IBM, Shanghai, China) were utilized to carry out preliminary statistical and differential testing of soil physicochemical properties and bacterial communities. The Chao1 estimator [59] and the ACE (Abundance-based Coverage Estimator) [60], community richness, the Shannon diversity index [61], and the Simpson index [62] were used to indicate community diversity. Non-metric multidimensional scale analysis (NMDS) was used to investigate the differences between soil samples [58]. The Bar diagram and Heatmap map were used to express the species composition differences. A student’s *t*-test, which was performed by SPSS 22 software (IBM, Shanghai, China), was used to distinguish among the differences in soil physicochemical properties and soil bacterial community diversity between the two groups. Origin 9.1 (OriginLab, Northampton, MA, USA) and the R project (R Development Core Team, Vienna, Austria) were used to make charts and graphs [63].

### 2.5. Soil Bacterial Molecular Ecological Network Construction and Analyses

**Figure 2:**
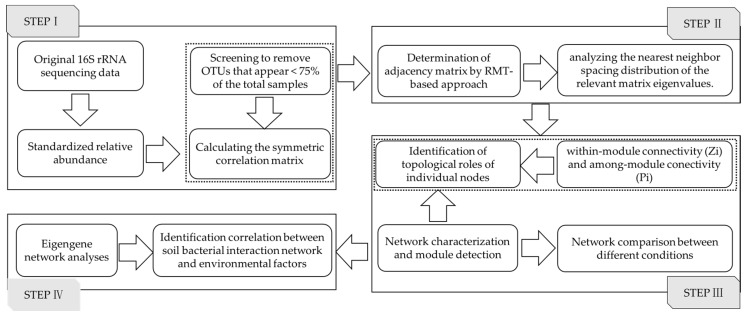
The framework of the soil bacterial molecular ecological network construction and analyses.

A soil bacterial molecular ecological network of coal mining cracks area, based on the Random Matrix Theory, was constructed by 16S rRNA sequencing data. Network construction and analysis were completed on the MENA (Molecular Ecological Network Analysis Pipeline, http://ieg4.rccc.ou.edu/mena) platform [39,40,43,51]. The Construction and Analyses steps of the molecular ecological network were as follows (Figure 2).

Step I: Original 16S rRNA sequencing data processing [40]. At 97% sequence identity, screening was done to remove OTUs (Operational Taxonomic Units) that appeared less than 75% of the total samples, and a more frequent occurrence of the OTUs distribution matrix was obtained. During the data preparation, the OTUs’ majority was kept, with eight in a total of 15 samples, and blanks were filled with 0.01 with paired valid values. Then, the obtained OTUs were converted with log10 and the symmetric correlation matrix was calculated. Then, the similarity matrix was transformed using the Pearson correlation coefficient.

Step II: Determination of the adjacency matrix by the RMT (random matrix theory) approach [41]. Based on the RMT method, the adjacency matrix was derived from the similarity matrix by selecting the appropriate threshold when there were significant non-random patterns at *p* < 0.05. The similarity threshold was set to 0.81 for the comparability between the two groups. The calculation order decreased the cutoff from the top using the regressed Poisson distribution. The connection strength between each pair of nodes was encoded by the adjacency matrix, and the ecological community was predicted by analyzing the nearest neighbor spacing distribution of the relevant matrix eigenvalues.

Step III: Network characterization and module detection. In MENA, a module in the network is a group of OTUs that are highly connected among themselves but have much fewer connections with OTUs outside the group [43]. The network was divided into several modules, each of which was a functional unit of the microbial system. Moreover, the network topological nodes and other related module information were obtained. The network node characteristics of soil bacterial communities were divided into the following four types by within-module connectivity (*Zi*) and among-module conectivity (*Pi*): (a) if *Zi* > 2.5 and *Pi* > 0.62, they were designated as network hubs; (b) if *Zi* > 2.5 and *Pi* ≤ 0.62, they were designated as module hubs; (c) if *Zi* ≤ 2.5 and *Pi* > 0.62, they were designated as connectors; and (d) if *Zi* ≤ 2.5 and *Pi* ≤ 0.62, they were designated as peripherals. In ecology, peripheral nodes are specialists, module hubs and connectors are similar to generalists, and network hubs represent supergeneralists [64,65]. Finally, using Cytoscape 3.7.1 software, the soil bacterial molecular ecological networks were visualized [66].

Step IV: Eigengene network analyses. Based on the above network module, the environmental factors were introduced to analyze the Module-EigenGene, and the correlations between the soil bacterial interaction network and environmental factors were analyzed using the Pearson correlation analysis and Mantel Test analysis [58].

## 3. Results

### 3.1. Effects of Coal Mining Cracks on Soil Physicochemical Properties

The effects of coal mining cracks on soil physicochemical properties are shown in Table 1. There were different changes in soil physicochemical properties in LC and CLC. Soil water, EC, and nitrate nitrogen did not show significant differences between LC and CLC (*p* > 0.05). The soil temperature dropped by about 5 °C in LC with a significant trend (*p* < 0.001). pH values decreased by about 0.4, and the downward trend was significant (*p* < 0.05); the soil showed weak acidity after coal mining cracks. The soil total salt content was reduced after the coal mining cracks. The OM decreased by about 1.5 g·kg^−1^ after coal mining cracks, and the decreasing trend was significant (*p* < 0.05). The soil AP decreased by about 40 mg·kg^−1^, and the decreasing trend was significant *(p* < 0.01). The soil AK decreased by about 85 mg·kg^−1^, and the downward trend was significant (*p* < 0.05). The soil ammonium nitrogen, which was different from the changes in other soil physicochemical properties, increased by about 0.5 mg·kg^−1^ with an extremely significant increasing trend (*p* < 0.001). From the point of view of space-time interchange, after the formation of coal mining cracks, the soil temperature, pH, OM, AP, and AK showed decreasing trends, but the AN showed an increasing trend.

### 3.2. Effects of Coal Mining Cracks on Soil Bacterial Community Structures

#### 3.2.1. Effects of Coal Mining Cracks on Soil Bacterial Community Diversity

**Figure 3:**
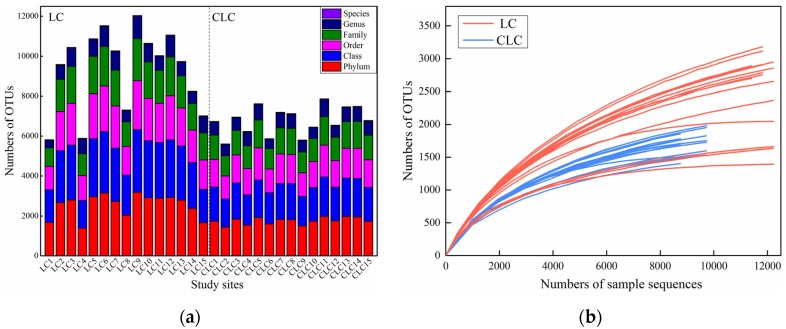
Statistics of OTUs (Operational Taxonomic Units) division and classification status appraisal (**a**) and rarefaction curve (**b**) in LC and CLC.

The statistics of the OTU division and classification status appraisal in LC and CLC are shown in Figure 3a. In this study, a total of 711,538 effective sequences and 496,862 OTUs were obtained in LC. A total of 690,569 effective sequences and 394,704 OTUs were obtained in CLC. Coal mining cracks showed more OTUs than the control areas. The rarefaction curve, a tool used to judge whether the current sequencing depth, which was sufficient to reflect the real bacterial diversity in the special region’s communities, is shown in Figure 3b. It was found that both the numbers of OTUs in LC and CLC tended to plateau and be almost flat in the case of saturation, which could indicate good coverage of the current soil bacterial community structure diversity. Moreover, the rarefaction curve of LC was longer and the sequencing quantity was higher than that of CLC.

**Figure 4:**
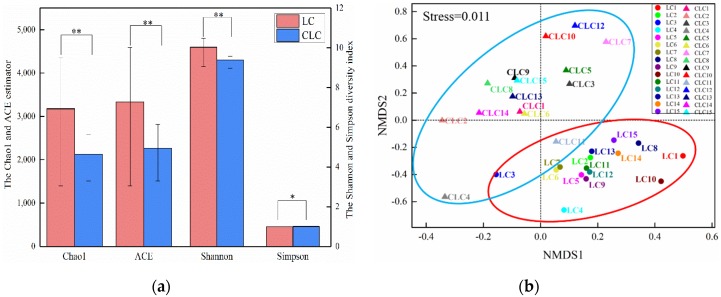
Alpha diversity index (**a**) and non-metric multidimensional scale analysis (NMDS) analysis (**b**) in the LC and CLC. (Note: * *p* < 0.05 indicates that the results of the univariate variance of LC and CLC were significant; ** *p* < 0.01 indicates that the results of the univariate variance of LC and CLC were very significant; the *p* value represents the significance of the *T*-test.).

The soil bacterial alpha diversity in the LC and CLC of Dongping Coal Mine are shown in Figure 4a. It is interesting that the diversity was increased in the coal mining cracks compared to the controls. Compared with the CLC, the soil bacterial community richness was increased in the coal mining cracks by about 50%. The Chao1 estimator and ACE estimator in the LC were higher than in the CLC (*p* < 0.01). Moreover, the soil bacterial uniformity increased in the coal mining cracks. The Shannon diversity index in the LC was higher and significantly changed compared with that in the CLC (*p* < 0.01), while the Simpson diversity index in the LC was smaller than that in the CLC (*p* < 0.05). Based on the Bray–Curtis similarity distance, an NMDS analysis of the soil bacterial community in the LC and CLC was carried out (Figure 4b). It showed that there were significant differences in the compositions of soil bacterial communities between the LC and CLC (stress < 0.05). However, there were also intersecting relations, which partly showed that there was a certain relationship between the LC and CLC.

#### 3.2.2. Effects of Coal Mining Cracks on the Soil Bacterial Community Composition

**Figure 5:**
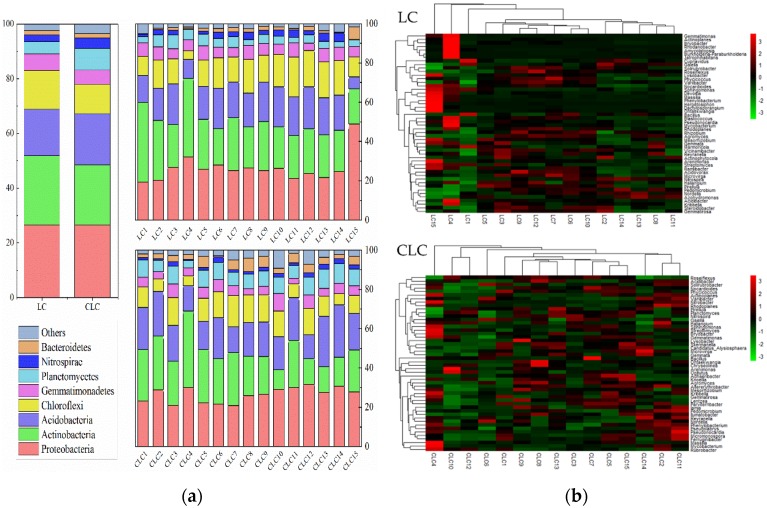
Bar analysis at the phylum level (**a**) and heatmap combined with cluster analysis at the genus level (the top 50) in the LC and CLC (**b**).

At the phylum level, the main bacterial species and the relative abundance of each sample were analyzed (Figure 5a). The soil bacterial phyla in the LC and CLC of Dongping Coal Mine accounting for more than 90% included *Proteobacteria*, *Actinobacteria*, *Acidobacteria*, *Chloroflexi*, *Gemmatimonadetes*, *Planctomycetes*, *Nitrospirae*, and *Bacteroidetes*. Furthermore, it has been found that the relative abundances of *Actinobacteria* and *Chloroflexi* were higher in the LC than in the CLC, but the abundances of *Planctomycetes*, *Bacteroidetes*, and *Proteobacteria* decreased.

Furthermore, the top 50 genera in the coal mining cracks area and the control area of the Dongping Coal Mine were clustered. The heatmap is shown in Figure 5b. Red indicates that the abundance of the coal mining cracks area and the control area were relatively high, and green indicates a relatively low abundance. The main genera of soil bacteria in the coal mining cracks area were different from in the control area. The top 10 genera in the coal mining cracks area were *Solirubrobacter*, *Cupriavidus*, *Roseiflexus*, *Lysobacter*, *Gaiella*, *Variibacter*, *Nocardioides*, *Phycicoccus*, *Haliangium*, and *Pirellula*, respectively. The top 10 genera in the control area were *Nocardioides*, *Solirubrobacter*, *Phycicoccus*, *Roseiflexus*, *Actinoplanes*, *Acidibacter*, *Variibacter*, *Gaiella*, *Pirellula*, and *Nitrobacter*, respectively. *Cupriavidus*, *Haliangium*, and *Lysobacter* appeared as the dominant genera in the coal mining cracks area. The top 50 genera with the highest relative abundance of the coal mining cracks area were from nine phyla, but the top 50 genera with the highest relative abundance from the control area were from 10 phyla. The genera belonging to Proteobacteria increased from 19 to 23 in the control area. At the level of genus, soil bacteria showed more diversity and variability.

### 3.3. Effects of Coal Mining Cracks on Soil Bacterial Community Interactions

#### 3.3.1. Effects of Coal Mining Cracks on the Topological Properties of the Soil Bacterial Molecular Ecological Network

The topological properties of soil bacterial molecular ecological networks are shown in Table 2. The R square values of the power-law in the LC and CLC were both bigger than 0.8, which indicates that both networks reflect the basic characteristics of the network without scale, small world, or modularization. There were 158 nodes and 273 links in LC and 260 nodes and 388 links in CLC. The average degree in LC was 3.456, which was a bit bigger than that of CLC. The average clustering coefficient in LC was 0.209, which was larger than that of CLC. The average path distance in LC was smaller than that of CLC. The geodesic efficiency in the LC was higher than that in the CLC. The maximal degree in the LC was almost the same as that of the CLC. The density was smaller in the CLC. The transitivity in the LC was bigger than that of the CLC. The connectedness in the LC was slightly higher than that of the CLC. The modularity showed the resistance characteristics of the network system against external changes, which was smaller in the LC but higher in the CLC. It was found that the soil bacterial molecular ecological network connection in the LC was more complex, the connection strength of the node and its adjacent nodes was enhanced, and the measurement efficiency was higher, but the transmission was weaker, and the resistance to the external changes was smaller.

#### 3.3.2. Effects of Coal Mining Cracks on the Topological Roles of Soil Bacterial Molecular Ecological Network Nodes

**Figure 6:**
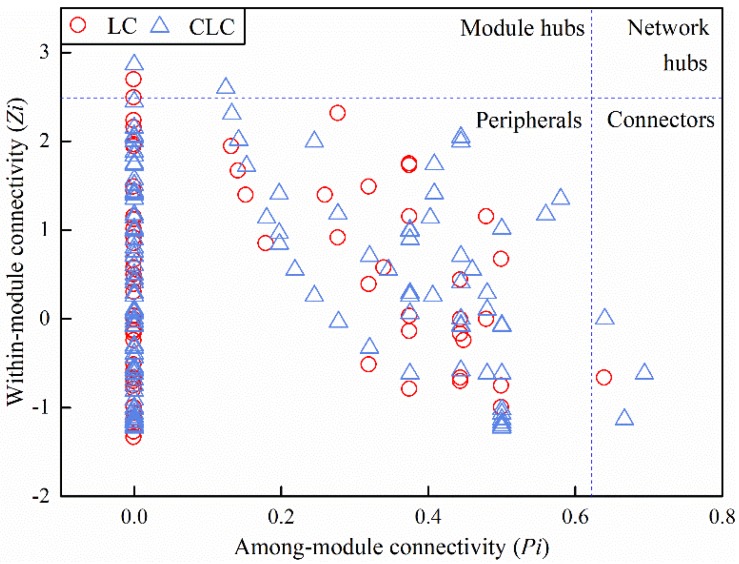
*Z-p* plot of molecular ecological networks in the LC and CLC. The *Z-p* plot shows the distribution of OTUs based on their topological roles. Each symbol represents an OTU under LC (red dot) or CLC (blue triangle). The peripherals have only a few links and almost always to the species within their modules; module hubs are highly connected to many species in their own modules; connectors are highly linked to several modules; network hubs have the function of module hubs and connectors.

The topological roles of soil bacterial molecular ecological network nodes in LC and CLC are shown in Figure 6. There was only one module hub in the LC, which belonged to Chloroflexi (OTU55051), whereas there were two module hubs in the CLC, both of which belonged to Actinobacteria, including OTU18780 and OTU72419. Moreover, there were three connectors in the CLC: OTU4303 belonged to Bacteroidetes, OTU29382 belonged to Acidobacteria, and OTU79476 belonged to Actinobacteria. There was only one connector in the LC: OTU6612 belonged to Proteobacteria. However, the network hub was not observed in either LC and CLC. It was found that land cracks after mining disturbance changed the network topology, individual OTU topological properties, and important microbial groups.

#### 3.3.3. Soil Bacterial Molecular Ecological Network Analysis in the LC and CLC

**Figure 7:**
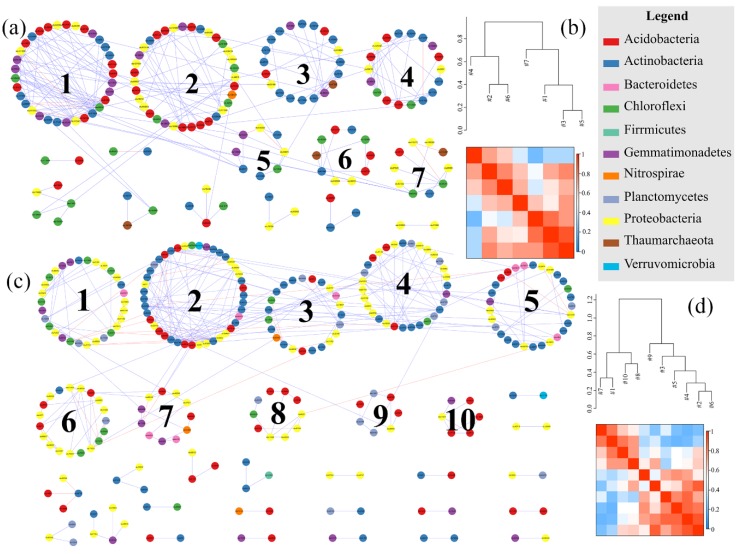
Soil bacterial molecular ecological networks and modular eigengene hierarchy structure in the LC and CLC. (**a**) Soil bacterial molecular ecological network in the LC; (**b**) soil bacterial modular eigengene hierarchy structure in the LC; (**c**) soil bacterial molecular ecological network in the CLC; (**d**) soil bacterial modular eigengene hierarchy structure in the CLC. Each circle represents an OTU. Different colors represent different phyla. Blue represents a positive interaction between two nodes, and red represents a negative interaction. The module was set with more than five nodes. The modular eigengene hierarchy structure indicates the Pearson correlation between the two modules.

The soil bacterial molecular ecological networks in the LC and CLC of Dongping mine are shown in Figure 7. Each node represents an OTU, and the different colors of the nodes represent different bacterial phyla. Blue represents a positive interaction between two nodes, and red represents a negative interaction. The positive and negative interactions of the soil bacterial molecular ecological networks in LC were about two times higher than those in CLC, but the roles of each OTU tended to cooperate with each other. In LC, Soil bacterial molecular ecological network was mainly composed of seven kinds of phyla, including *Acidobacteria*, *Actinobacteria*, *Chloroflexi*, *Gemmatimonadetes*, *Nitrospira*, *Proteobacteria*, and *Thaumarchaeota*. However, there were nine kinds of phyla, including *Acidobacteria*, *Actinobacteria*, *Bacteroidetes*, *Chloroflexi*, *Firmicutes*, *Gemmatimonadetes*, *Nitrospira*, *Planctomycetes*, *Proteobacteria* and *Verrucomicrobia* in the CLC network. Soil bacterial communities’ structure and interactions were complexed after coal mining cracks. Soil bacterial molecular ecological network in LC mainly formed seven modules, while there were 10 modules in CLC. There was also a close connection between different modules. In LC, all modules linked closely. In CLC, from module 1 to module 7, the modules were closely linked, while module 8, module 9, and module 10 were not linked very closely to other modules. It was found that coal mining cracks strengthened the interactions among soil bacteria.

### 3.4. Effects of Coal Mining Cracks on the Relationship between Soil Bacterial Communities and Physicochemical Properties

In order to demystify the effects of coal mining cracks on the relationship between soil bacterial communities and physicochemical properties, the Mantel Test was analyzed under the OTU level, and the results are shown in Table 3. The results show a significant relationship between soil bacterial communities and soil temperature (*p* < 0.01), EC (*p* < 0.01), and nitrate nitrogen (*p* < 0.01) in LC. The soil pH value showed a statistically significant relationship with soil bacterial communities in the LC (*p* = 0.05), while the soil pH was significantly correlated with soil bacterial communities in the CLC (*p* < 0.05).

**Figure 8:**
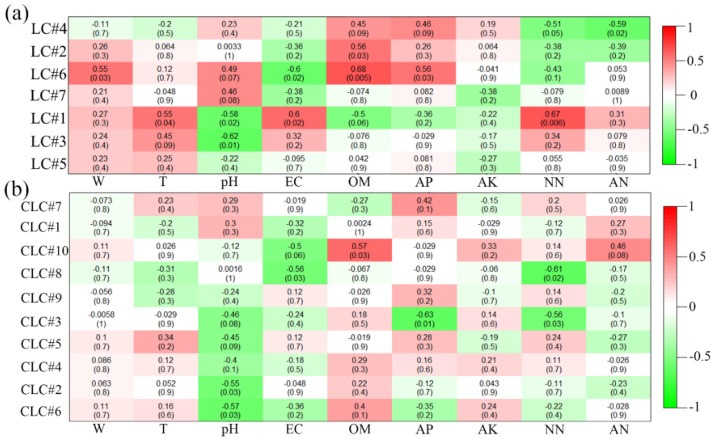
Heatmap analysis of module correlations with environmental factors in the LC (**a**) and CLC (**b**).

Furthermore, at the OTU level, a heatmap analysis of each module gene and environmental factor was conducted, and the results are shown in Figure 8. In LC, the results showed that the OTU of the cluster in module 1 was significantly correlated with T, EC, and NN. The OTU of clustering in module 6 was significantly correlated with W, OM, and AP, while module 2 was significantly correlated with OM. The pH value was negatively correlated with modules 1 and 3. The EC was negatively correlated with module 6, and the AN was negatively correlated with module 4. On the other hand, in the CLC, only module 10 was positively correlated with the OM. The pH was negatively correlated with module 2 and module 6. The EC was negatively correlated with module 8, and the AP was negatively correlated with module 3. Module 3 and module 8 were also negatively correlated with the NN. Constructed with LC and CLC, the soil physicochemical properties had different changes with related modules. The EC changed from a positive correlation to a negative correlation. The relationship between the soil temperature and modules changed to significant in the LC, while the AP was changed to a significant correlation. The W showed a significant correlation in the LC. The significant positive correlation between OM and modules did not change too much.

## 4. Discussion

In this study, it was found that soil physicochemical properties and bacterial communities undergo some changes due to the pressure of coal mining cracks.

Due to coal mining cracks, most of the soil physicochemical properties were found to decrease, similar to the results of some previous studies [22,23,24,25,26,27]. The soil water holding capacity and heat-retaining property reduced, which might be due to increased airflow rate and moisture evaporation from the cracks [67]. The soil showed weak acidity in the coal mining cracks area. The contents of total salt quantity and organic matter were reduced, which might have been caused by the loss of soils and residues [68]. The elements of phosphorus and potassium were lost, which might have affected the growth of the surrounding plants [69]. It was interesting that the concentration of nitrate nitrogen was decreased but that of ammonium nitrogen was increased. The nitrate ion is a negatively charged anion that cannot be absorbed by soil colloids and is easily lost with water. In contrast, the ammonium ion has a positive charge and is easily absorbed by the soil colloid and is not easily lost [70]. These different changes in soil physicochemical properties provide a good reference for the remediation of soil degradation for coal mining cracks.

The characteristics of soil bacterial community structures and interactions were strengthened after coal mining cracks. In this study, the relative abundances of *Actinobacteria* and *Chloroflexi* increased, while those of *Planctomycetes*, *Bacteroidetes*, and *Proteobacteria* decreased. It was also found that the sequencing quantity and diversity of the soil bacterial community increased obviously. Those different changes indicate that coal mining cracks have an enormous effect on the soil microenvironment [28,30]. Moreover, there were fewer modules after coal mining cracks, but the interactions between different bacterial communities were closer. It has been observed that seven modules were formed after coal mining cracks, which reduced three modules than the control area. However, the links among these modules were significantly strengthened. This might indicate that bacterial communities were more inclined to cooperate with each other to adapt to the mutated environment, and they had similar niches during the formation of cracks after coal mining disturbance [39,55]. Furthermore, some region-specific OTUs were found in Dongping Coal Mine. Only one module hub (OTU5505, *Chloroflexi*) and one connector (OTU6612, *Proteobacteria*) formed after the development of coal mining cracks. In the unaffected control areas, there were two module hubs (OTU18780, *Actinobacteria*, and OTU72419, *Actinobacteria*) and three connectors (OTU4303, *Bacteroidetes*; OTU29382, *Acidobacteria*; and OTU79476, *Actinobacteria*). These unknown OTUs might be the focus of future research.

Coal mining cracks destroy the balance of original soil living conditions [6,30]. Some soil physicochemical properties show a close relationship with bacterial communities. It has been found that soil moisture deficiency stress is serious after the coal mining cracks [24,67]. In this study, the soil water content was identified as the main factor affecting the diversity of soil bacterial community structure, similar to previous reports on the effects of water stress on soil bacterial communities [55,71,72]. Moreover, the pH was also found to be an important factor affecting the diversity of soil bacterial communities, a result that was also similar to other studies [73,74,75,76,77,78]. Previous experiments have illustrated that pH is a good predictor of spatial distribution at Changbai Mountain [74], in the Arctic [73,78], at the continental-scale [75], and on the global atlas [77]. However, no significant relationships between soil bacterial communities and other soil physicochemical properties were identified. The negative correlation between nitrate nitrogen and effective phosphorus changed to a positive correlation from CLC to LC, which explained the potential changes in the soil microbial community structure after soil nutrient loss. The relationship changes between the bacterial species and environmental factors corresponding to the OTUs in these potential feature modules would also provide a powerful tool for clarifying their responses to the bacterial community structure diversity and interactions in the event of environmental change, which would be helpful for the development of new strains to facilitate the closure of cracks.

However, we realize that our research might have two limitations. The first is that this study did not cover the differences in soil bacterial communities under pressure with changes of years. This limitation was due to the difficulty of collecting long time-series data of cracks after a coal mining disturbance. Under the double pressure of environmental supervision and public opinion, the coal mine enterprises and local government have carried out timely reclamation activities on coal mining cracks [5]. The second limitation was that the relationship between vegetation diversity and bacterial communities was not considered. Previous studies have shown that the relationship between microorganisms and vegetation is close [79,80,81]. Different types of covering vegetation would affect the diversity and interactions of microorganisms [76,80]. This study only controlled a small amount of grass in the coal mining cracks, but there was no consideration of the specific classification of grass species. Further studies, which should take interannual variations and vegetation differences into account, need to be performed.

Furthermore, in this study, the soil bacterial molecular ecological network was introduced as an interesting tool to characterize the changes in bacterial communities’ interactions [40,43,51]. From observing the changes in modules before and after coal mining cracks, it was indicated that soil bacterial communities have undergone adaptive cooperation. It is expected that the changes in bacterial community network interactions could be used as a new indicator to monitor the changes in the ecological environment in mining areas.

## 5. Conclusions

This study investigated the changes in soil bacterial communities in a post-mining ecosystem and demonstrated that cracks reinforced the interactions among soil bacterial communities in the coal mining area of the Loess Plateau, China. Some important observations are summarized below.

First, there was a partial loss of soil nutrients in the post-mining cracks area. The content of soil water, temperature, pH, EC, OM, available phosphorus, and available potassium reduced, while the content of ammonium nitrogen was increased. Second, the soil bacterial community structure richness and uniformity increased by about 50% in the post-mining cracks area. The phyla representing more than 90% of all phyla were *Proteobacteria*, *Actinobacteria*, *Acidobacteria*, *Chloroflexi*, *Gemmatimonadetes*, *Planctomycetes*, *Nitrospirae*, and *Bacteroidetes*. Moreover, the soil bacterial molecule ecological network connection tended to be more complicated in the post-mining cracks area. The topological attributes of the individual OTUs and the important microbial groups changed obviously. The relationship between network connections and interactions was strengthened. Third, the difference between the soil bacterial community structure composition was caused by soil nutrient loss due to coal mining cracks. Furthermore, the relationship between soil physicochemical properties and modules changed in a complex manner. This study also observed that there were significant relationships among soil bacterial communities and the soil organic matter content and the pH value.

This study provides a reference for the treatment of cracks in the Loess Plateau mining area and similar regions around the world and provides new ideas for the environmental changes caused by human activities.

## Figures and Tables

**Table 1:** Characteristics of soil physicochemical properties in LC and CLC.

Soil Physicochemical Properties	LC	CLC	*p* ^2^
Soil water/W (%)	15.60 ± 2.45 ^1^	17.35 ± 5.22	0.266
Soil temperature/T (°C)	21.24 ± 0.71	26.14 ± 2.98	<0.001 ***
pH	7.20 ± 0.30	7.64 ± 0.37	0.002 **
Electrical conductivity/EC (ms·cm^−3^)	13.83 ± 8.68	16.13 ± 6.92	0.446
Organic matter/OM (g·kg^−1^)	2.64 ± 0.72	4.37 ± 2.65	0.026 *
Available phosphorus/AP (mg·kg^−1^)	107.04 ± 35.67	147.49 ± 40.00	0.009 **
Available potassium/AK (mg·kg^−1^)	69.00 ± 32.78	165.57 ± 131.80	0.013 *
Nitrate nitrogen/NN (mg·kg^−1^)	1.68 ± 2.21	3.37 ± 2.67	0.08
Ammonium nitrogen/AN (mg·kg^−1^)	1.23 ± 0.27	0.76 ± 0.17	<0.001 ***

Note: ^1^ Average value ± Standard deviation of the coal mining cracks area and the control area; ^2^ *p* valve represents the significance of *T*-test; * *p* < 0.05: significant; ** *p* < 0.01: very significant; *** *p* < 0.001: extremely significant.

**Table 2:** Comparison of the topological properties of soil bacterial molecular ecological networks in the LC and CLC in Dongping Coal Mine.

Molecular Ecological Network Topological Properties	LC	CLC
Number of original OTUs ^1^	469	448
Similarity threshold ^2^	0.81	0.81
Total nodes ^3^	158	260
Total links ^4^	273	388
R square of power-law ^5^	0.824	0.926
Average degree ^6^	3.456	2.985
Average clustering coefficient ^7^	0.209	0.155
Average path distance ^8^	5.663	6.608
Geodesic efficiency ^9^	0.230	0.200
Maximal degree ^10^	14	15
Density ^11^	0.022	0.012
Transitivity ^12^	0.328	0.274
Connectedness ^13^	0.744	0.611
Module ^14^	15	31
Modularity ^15^	0.687	0.710

Note: ^1^ The number of original OTUs refers to the number of OTUs participating in building the network; ^2^ The similarity threshold was set to allow comparison where the significant non-random patterns were both at *p* < 0.05. ^3^ Total nodes represents the number of nodes formed by the network construction. ^4^ Total links represents the number of connections formed by the network construction. ^5^ The R square of power-law represents the credibility of scale-free networks. ^6^ Average degree indicates the strength of connectivity between different OTUs. ^7^ The average clustering coefficient was used to measure the extent of module structure present in a network. ^8^ The average path distance indicates the average distance between two nodes. ^9^ The geodesic efficiency was used to characterize the closeness between nodes. ^10^ The maximal degree is the maximum number of connections between a node and other nodes. ^11^ The density indicates the complexity of the network. ^12^ The transitivity is the entire clustering coefficient. ^13^ Connectedness is one of the most important measurements for summarizing hierarchical structures. ^14^ A module in the network is a group of OTUs that are highly connected among themselves but have much fewer connections with OTUs outside the group. ^1^^5^ Modularity was used to measure the quality of community segmentation [43].

**Table 3:** Mantel Test between the soil microbial community on the OTU level and environmental factors in the LC and CLC.

Soil Physicochemical Properties	LC		CLC	
	*Mantel r*	*p*	*Mantel r*	*p*
W (%)	−0.0934	0.999	−0.0087	0.485
T (°C)	**0.1578**	**0.001**	−0.0596	0.961
pH	0.0811	0.050	**0.0769**	**0.022**
EC (ms·cm^−3^)	**0.1803**	**0.003**	−0.0554	0.944
OM (g·kg^−1^)	0.0505	0.092	0.0126	0.340
AP (mg·kg^−1^)	−0.0086	0.486	−0.0455	0.875
AK (mg·kg^−1^)	−0.0339	0.722	−0.0332	0.743
NN (mg·kg^−1^)	**0.1790**	**0.003**	−0.0769	0.998
AN (mg·kg^−1^)	0.0356	0.198	−0.0180	0.607
